# Infectious disease ecology and evolution in a changing world

**DOI:** 10.1098/rstb.2022.0002

**Published:** 2023-03-27

**Authors:** Kayla C. King, Matthew D. Hall, Justyna Wolinska

**Affiliations:** ^1^ Department of Biology, University of Oxford, Oxford OX1 3SZ, UK; ^2^ School of Biological Sciences, Monash University, Melbourne 3800, Australia; ^3^ Department of Evolutionary and Integrative Ecology, Leibniz Institute of Freshwater Ecology and Inland Fisheries (IGB), 12587 Berlin, Germany; ^4^ Department of Biology, Chemistry, and Pharmacy, Institute of Biology, Freie Universität Berlin (FU), 14195 Berlin, Germany

## 1. Introduction

Managing the consequences of climate change and human activity is one of the greatest challenges of the twenty-first century. Extreme weather events, such as heatwaves and droughts, are becoming more common and more severe [1], and urban and agricultural expansion is contributing to the loss of biodiversity and the degradation of ecosystems [2]. This new reality challenges the capacity of host species to persist and forces infectious diseases to rapidly evolve. Indeed, the COVID-19 pandemic has emphasized how quickly infectious diseases can evolve and spread—with consequences for transmission, virulence and evasion of host defences [3]—and that disease dynamics will play out differently across regions of the globe [4,5].

Addressing these challenges requires multi-faceted approaches that explore the effects of human-induced change on host–pathogen (including parasites) interactions across space and time (i.e. ecological and evolutionary timescales). Developing measures to enhance the resilience of natural and agricultural communities to these changes is also critical. Yet, these concepts have traditionally been tackled from disparate viewpoints with little empirical overlap. Ecological and epidemiological research, for example, has linked the spread of disease to environmental factors (e.g. [6–8]). But there is a pressing need for evolutionary research to capture how hosts and their pathogens may evolve under the sweeping environmental changes that populations now face. There is evidence that abiotic environmental factors can impact selection in host–pathogen interactions (e.g. [9]), but often empirical work examines these questions in abstract ways (hot versus cold, low versus high food). In turn, suggestions to use ecological and evolutionary principles in the management of agricultural pests and pathogens do not often consider the social and economic factors that underlie any long-term intervention [10].

This special issue aims to bring together research from different fields (ecological, evolutionary, epidemiological and applied) and approaches to better understand and address the impact of human-driven environmental change on infectious disease. It addresses a lack of comprehensive discussion on key issues that arise because different types of environmental change often form their own fields of research, rather than being studied as interconnected symptoms of human activity. To address this gap, the contributions herein focus on three key themes: *climate change and infection outcomes*; *understanding host–pathogen interactions in dynamic environments*; and *outbreaks and pathogen evolution in human-altered ecosystems*. By comparing different forms of global change, integrating across multiple fields, and identifying empirical and theoretical research gaps, this issue's goal is to showcase and spark new thinking on infectious disease evolution in a rapidly changing world.

## 2. Theme 1: climate change and infection outcomes

Thermal conditions can strongly impact host- and pathogen infection-related traits. Temperature has been shown to alter the encounter rates between host and parasites, host susceptibility and tolerance to infection and, finally, the infectivity and virulence of parasites (reviewed in [11–14]). There is a common prediction of increased pathogen development and replication rates as well as enhanced parasite transmission under elevated temperatures (i.e. ‘warmer hence sicker world’ scenario, e.g. [12]). However, the empirical evidence is inconclusive—certain studies support but others contradict (e.g. [15–17]) this hypothesis.

The first group of papers focus on the most widely considered facet of global change—climate change and rising temperatures—but address the pressing need to consider these in the light of host and pathogen traits as well as their spread and evolutionary potential. Empirical studies explore the direct impacts of warming on key pathogen infectivity and host resistance traits, with implications for disease outbreaks. A field study extends exploration of trait variation in the larger host community to disease risk as temperature increases. An empirical study and an opinion piece further consider the roles host and pathogen adaptation play in climate-driven disease emergence and severity. Whether hosts can shift their thermal tolerance, or pathogens evolve to become more virulent, has major implications for species persistence in a warming world.

To better understand the impact of environmental temperatures on disease outbreaks, two papers focus on changes in mechanisms underpinning the ability of parasites to infect [18] and of hosts to resist [19]. In effect, these papers take care to start from the beginning, where the host and parasite first meet, using the planktonic crustacean *Daphnia* and its bacterial parasites. Marcus *et al*. [18] found that parasite spores exposed to higher temperatures were impaired in their ability to attach to hosts and subsequently establish infection. The degree of impact on these traits also varied depending on whether spores were desiccated or kept wet during heat spells. It is not the case that a warmer world is sicker if parasite survival is at risk between hosts. Like most hosts, waterfleas have physical barriers to infection and also have cellular immune responses. In detailed experiments, Sun *et al*. [19] found that warming affected these defence traits differently, with consequences for infection outcomes. Disease spread can also depend on how temperature interacts with traits in host communities [20,21]. In a thorough field study set in the Swiss Alps, Halliday *et al*. [22] found– complex relationships between trait variation in plant communities driven by temperature gradients and prevalence of infection in those communities. Together, these three studies highlight that to predict disease dynamics under climate change, we should consider multiple traits at the interface of host–pathogen interactions and their variation within species and communities.

The final two papers in this theme address the evolutionary potential of hosts [23] and pathogens [24] in the face of rising temperatures. The geographical ranges of hosts, and the pathogens they carry, are expanding owing to changing climates [25]. Of particular concern is the future impact of climate change on the potential for mosquitoes—vectors for many human disease-causing viruses—to adapt to shifts in their thermal environment and move into new areas. Ware-Gilmore *et al*. [23] thoughtfully address this issue by studying how the heritable genetic variation and physiological responses in the mosquito *Aedes aegypti* may affect the upper thermal limits in populations over evolutionary time. In addition to driving vector evolution, will climate change select for more virulent pathogens? In the light of the classic virulence–transmission trade-off [26], Hector *et al*. [24] discuss what happens to pathogen virulence, burdens, and replication in host populations suffering heat stress. The authors find predicting the evolution of pathogen virulence amidst climate change might require a better understanding of transmission strategies and covariation among pathogen traits.

## 3. Theme 2: understanding host–pathogen interactions in dynamic environments

The study of abrupt environmental changes, such as hot versus cold or pristine versus polluted (e.g. see [9,27,28]) is often used to interpret the ecological or evolutionary impact of global change. However, the varied and dynamic nature of human-induced change is highly realistic. The simplification of environmental change is understandable given the added sample sizes that studying multiple host or pathogen genotypes or species necessitates. Yet, this situation is rapidly changing. For the study of thermal change, for example, a range of temperatures are increasingly being used to study thermal performance curves [8,29] or even the variation in daily thermal fluctuations or heatwaves [30]. New theoretical and empirical approaches, however, are now needed to expand our capacity to quantify the dynamics of environmental change and predict host and pathogen evolution in nature.

The first two papers in this theme evaluate how well current modelling approaches perform in capturing the rapidly changing environments that host and pathogens face. Best & Ashby [31] review the main approaches used to model host–pathogen evolution when ecological dynamics fluctuate owing to either extrinsic (seasonality, food availability) or intrinsic (time lags) factors. They then provide an in-depth guide on how to implement one main method and apply this approach to fluctuations arising from seasonally varying resources, among others. By contrast, Jiranek *et al.* [32] review the use of mechanistic models to study host–pathogen interactions under different scenarios of climate change, with a focus on plant systems. They outline the challenge of linking disease outbreaks with weather variables when climate change will likely affect many aspects of host and pathogen physiology, host demography, and pathogen life cycles, and these effects may frequently be nonlinear. The authors then discuss how mechanistic models overcome this limitation. These models can leverage data from wild and agricultural plant–pathogen systems to understand the complex feedback loops arising among physiological, demographic and evolutionary processes.

Complementing the modelling-focused perspectives are two empirical papers exploring how local environmental conditions predict disease prevalence, severity and evolution. Graham *et al.* [33] highlight the utility of using high-throughput phenotyping to make disease comparisons across large environmental, spatial and temporal gradients. By surveying seagrass wasting disease in eelgrass meadows throughout their northern range (covering eight degrees of latitude) they show that disease prevalence and severity was lower in cooler sites, colder years, and higher latitudes. The authors provide several suggestions for how this new information can improve eelgrass management. The final paper in this theme, by Melero *et al.* [34], considers how climate change and human activity might induce changes in plant development that can shape the evolution of host–virus interactions. Using *Arabidopsis thaliana* as a model system, the authors experimentally evolved a turnip mosaic virus at three different host developmental stages. They found hosts in later developmental stages were prone to faster and more severe infections, but the virus nonetheless evolved more rapidly in younger hosts.

## 4. Theme 3: outbreaks and pathogen evolution in human-altered ecosystems

The third group of papers considers the broader extent of direct human activity on host–pathogen interactions. Synthesis and empirical papers explore the socio-economic challenges of implementing evolutionarily responsive practices in agriculture, and the consequences for pathogen evolution when an intervention, such as well-meaning habitat restoration, goes wrong. Geffersa *et al*. [35] summarize research efforts on crop disease management based on deployment of resistance genes. The goal is to disrupt pathogen adaptation and prevent the breakdown of resistance. However, practical uptake of such strategies is limited, and applied evolutionary research to control pathogen adaptation can have socio-economic challenges. Geffersa *et al*. develop a conceptual framework for the economic valuation of engineering of genes conferring resistance, emphasizing the value of these strategies beyond economic benefits. Feau *et al*. [36] argue that the introduction of new host species can accelerate pathogen evolution and affect long-established host–pathogen coevolutionary dynamics. Specifically, the emergence of a new pathogen lineage with the intensification of poplar tree cultivation causes stem infections in a new host. This represents a serious threat to poplars and could affect both natural and planted forests. Finally, Manley *et al*. [37] explore how conservation measures to protect pollinators—planting wildflowers along fields—affect disease prevalence in pollinator communities. They found wildflower patches did not act as transmission hubs, but reduced the prevalence of some viral infections, playing an unintended but additional role in pollinator conservation.

Complementing these papers is a novel empirical exploration of the consequences of emerging pollutants, namely nanoplastics [38] and pharmaceuticals [39]. Unlike other well-studied pollutants, wastewater treatment is often inadequate in removing nanoplastics and pharmaceuticals. These pollutants will thus remain a problem in the coming decades. Plastic production is estimated to reach 33 billion tons by 2050 [40] and is particularly insidious because plastics break down into smaller particles called micro- or nanoplastics (size less than 5 mm and less than 100 nm, respectively) that can cross cell membranes, penetrate organs and bioaccumulate in organisms. In the current issue, Manzi *et al*. [38] and Aulsebrook *et al*. [39] both used the planktonic crustacean *Daphnia* as experimental host. Manzi *et al*. found that it depends on the type of parasite whether and to what extent nanoplastics affect the infection. Low plastic load had no direct negative consequences for the host, but infection rates either greatly increased (*Metschnikowia bicuspidata*) or were impeded (*Ordospora colligata*). These results indicate that distinct parasite species can show contradictory responses to a contaminant and that nanoplastics can favour co-infections.

Thousands of pharmaceuticals are used for health management in humans, pets and agricultural animals, and over 600 products have now been detected in the wild [41]. These pollutants remain bioactive when excreted, are often resistant to degradation, and target receptors conserved in many species [42–44]. Aulsebrook *et al*. [39] showed that the non-monotonic effects of fluoxetine were only expressed once a host was infected, demonstrating that the full impact of pharmaceuticals may only be experienced in the presence of other stressors. Parameterizing an epidemiological model, they further explore how fluoxetine can shape the likelihood of an infectious disease outbreak. Their result reiterates the findings of Manzi *et al*. [38] that the effects of pollutants are likely to be pathogen species or genotype specific. Both pollutants exemplify the complexity of modern human activity on disease dynamics by acting in a nonlinear (non-monotonic) manner and causing an unexpected exaggeration of infection outcomes at trace amounts.

## 5. Future directions and conclusion

The consequences of global change to infectious disease ecology and evolution are relevant for the health of humans, animals, plants and the environment. In this issue, climate change, environmental pollution and the increasing movement of people, animals or cultivars, are presented as examples of human-induced change that can affect the emergence and evolution of hosts, pathogens and vectors. Beyond public or ecosystem health concerns, pathogen spread and evolution due to global change have also been presented as intimately linked to issues of food security—crop production and other aspects of agriculture (including aquaculture and apiculture). Arising from the series of papers across the three themes of this special issue are these takeaways:

1. We need to know more about the potential and realized evolutionary paths of hosts and pathogens in a human-altered world. Both host and pathogen responses to climate change, emerging pollutants, or even interventions, are likely to be species or genotype specific. Pathogens and many pathogen-carrying invertebrates can also evolve on short timescales that are relevant for predicting disease outbreaks, as well as the likelihood and impact of zoonoses.
2. A warmer or heavily modified world is not always sicker. Temperature affects each component of host–pathogen interactions in unique ways, from host demography to within-host pathogen burden, making simple generalizations difficult. While traditional pollution at a greater dose is usually more damaging for hosts or pathogens, for emerging pollutants (i.e. pharmaceuticals), the greatest effects can often occur at the smallest doses, owing to the way these chemicals target conserved pathways.
3. Not all populations will be equally impacted by change. Host and pathogen populations should be expected to be adapted to their local environment, and disease outcomes will likely vary with latitude, altitude, water depth (in the case of aquatic organisms), or prior exposure to human activity. Local adaptation is key both to understanding how host and pathogen responses to human activity might vary over space and time, and for making predictions on the future distribution of vectors and pathogens under different change scenarios.
4. Opportunities to expand the scope with which human-induced change is studied are sorely needed. Directly incorporating fluctuating ecological dynamics into our studies or using empirical data to build mechanistic models of different types of change offer some solutions for predicting evolutionary change in response to human modification.
5. Parasitism is one of the most common lifestyles on Earth [45]. There is a need to incorporate interactions between host species and their pathogens as new ecotoxicological endpoints to better assess the ecological consequences of novel pollutants. Assessing the effects of any pollutant in isolation and, in particular, dismissing infection may lead to a severe underestimation of their real impact on individual host physiology, with upscaling effects on overall populations and ecosystems.
6. Adapting evolutionary or ecological principles into the management of human activity, pests or pathogens is not without its costs. The longer timescales with which these implementations operate, particularly when compared with traditional agricultural approaches, for example, create additional social and economic challenges that are often not appreciated when eco–evo ideas are first suggested.

Overall, this issue summarizes current progress and identifies remaining gaps in our understanding of infectious disease ecology and evolution in a global change framework. We hope the special issue will help drive new research on host–pathogen interactions, integrating traditionally isolated fields of study.

## Data Availability

This article has no additional data.

## References

[1] Mazdiyasni O, AghaKouchak A. 2015 Substantial increase in concurrent droughts and heatwaves in the United States. Proc. Natl Acad. Sci. USA **112**, 11 484-11 489. (10.1073/pnas.1422945112) PMC4577202 26324927

[2] Cardoso MC, Gonçalves RB. 2018 Reduction in half: the impact on bees of 34 years of urbanization. Urban Ecosyst. **21**, 943-949. (10.1007/s11252-018-0773-7)

[3] Day T, Gandon S, Lion S, Otto SP. 2020 On the evolutionary epidemiology of SARS-CoV-2. Curr. Biol. **30**, R849-R857. (10.1016/j.cub.2020.06.031) 32750338 PMC7287426

[4] Chen S, Prettner K, Kuhn M, Geldsetzer P, Wang C, Bärnighausen T, Bloom DE. 2021 Climate and the spread of COVID-19. Scient. Rep. **11**, 9042. (10.1038/s41598-021-87692-z) PMC8079387 33907202

[5] Rodó X, San-José A, Kirchgatter K, López L. 2021 Changing climate and the COVID-19 pandemic: more than just heads or tails. Nat. Med. **27**, 576-579. (10.1038/s41591-021-01303-y) 33820994

[6] Civitello DJ, Penczykowski RM, Hite JL, Duffy MA, Hall SR. 2013 Potassium stimulates fungal epidemics in *Daphnia* by increasing host and parasite reproduction. Ecology **94**, 380-388. (10.1890/12-0883.1) 23691657

[7] Cressler CE, Nelson WA, Day T, McCauley E. 2014 Disentangling the interaction among host resources, the immune system and pathogens. Ecol. Lett. **17**, 284-293. (10.1111/ele.12229) 24350974 PMC4264941

[8] Mordecai EA et al. 2019 Thermal biology of mosquito-borne disease. Ecol. Lett. **22**, 1690-1708. (10.1111/ele.13335) 31286630 PMC6744319

[9] Wolinska J, King KC. 2009 Environment can alter selection in host–parasite interactions. Trends Parasitol. **25**, 236-244. (10.1016/j.pt.2009.02.004) 19356982

[10] He DC, Burdon JJ, Xie LH, Jiasui ZH. 2021 Triple bottom-line consideration of sustainable plant disease management: from economic, sociological and ecological perspectives. J. Integr. Agric. **20**, 2581-2591. (10.1016/S2095-3119(21)63627-4)

[11] Harvell CD, Mitchell CE, Ward JR, Altizer S, Dobson AP, Ostfeld RS, Samuel MD. 2002 Climate warming and disease risks for terrestrial and marine biota. Science **296**, 2158-2162. (10.1126/science.1063699) 12077394

[12] Altizer S, Ostfeld RS, Johnson PTJ, Kutz S, Harvell CD. 2013 Climate change and infectious diseases: from evidence to a predictive framework. Science **341**, 514-519. (10.1126/science.1239401) 23908230

[13] Lafferty KD, Mordecai EA. 2016 The rise and fall of infectious disease in a warmer world. F1000Research **5**, 2040. (10.12688/f1000research.8766.1) PMC4995683 27610227

[14] Marcogliese DJ. 2008 The impact of climate change on the parasites and infectious diseases of aquatic animals. Rev. Sci. Tech. **27**, 467-484. (10.20506/rst.27.2.1820) 18819673

[15] Raffel TR, Michel PJ, Sites EW, Rohr JR. 2010 What drives chytrid infections in newt populations? Associations with substrate, temperature, and shade. Ecohealth **7**, 526-536. (10.1007/s10393-010-0358-2) 21125308

[16] Gehman A-LM, Hall RJ, Byers JE. 2018 Host and parasite thermal ecology jointly determine the effect of climate warming on epidemic dynamics. Proc. Natl Acad. Sci. USA **115**, 744-749. (10.1073/pnas.1705067115) 29311324 PMC5789902

[17] Dziuba MK, Manzi F, Cerbin S, Wolinska J. 2022 Can climate warming save *Daphnia* from parasites? Reduced parasite prevalence in *Daphnia* populations from artificially heated lakes. Limnol. Oceanogr. **68**, 181-191. (10.1002/lno.12257)

[18] Marcus E, Dagan T, Asli W, Ben-Ami F. 2023 Out of the ‘host’ box: extreme off-host conditions alter the infectivity and virulence of a parasitic bacterium. Phil. Trans. R. Soc. B **378**, 20220015. (10.1098/rstb.2022.0015) 36744562 PMC9900709

[19] Sun S-J, Dziuba MK, Jaye RN, Duffy MA. 2023 Temperature modifies trait-mediated infection outcomes in a *Daphnia*–fungal parasite system. Phil. Trans. R. Soc. B **378**, 20220009. (10.1098/rstb.2022.0009) 36744571 PMC9900708

[20] Rohr JR, Dobson AP, Johnson PTJ, Kilpatrick M, Paull SH, Raffel TR, Ruiz-Moreno D, Thomas MB. 2011 Frontiers in climate change–disease research. Trends Ecol. Evol. **26**, 270-277. (10.1016/j.tree.2011.03.002) 21481487 PMC3374867

[21] Lafferty KD. 2009 The ecology of climate change and infectious diseases. Ecology **90**, 888-900. (10.1890/08-0079.1) 19449681

[22] Halliday FW, Czyżewski S, Laine A-L. 2023 Intraspecific trait variation and changing life-history strategies explain host community disease risk along a temperature gradient. Phil. Trans. R. Soc. B **378**, 20220019. (10.1098/rstb.2022.0019) 36744568 PMC9900715

[23] Ware-Gilmore F, Novelo M, Sgrò CM, Hall MD, McGraw EA. 2023 Assessing the role of family-level variation and heat shock gene expression in the thermal stress response of the mosquito *Aedes aegypti*. Phil. Trans. R. Soc. B **378**, 20220011. (10.1098/rstb.2022.0011) 36744557 PMC9900713

[24] Hector TE, Gehman A-LM, King KC. 2023 Infection burdens and virulence under heat stress: ecological and evolutionary considerations. Phil. Trans. R. Soc. B **378**, 20220018. (10.1098/rstb.2022.0018) 36744570 PMC9900716

[25] Cohen JM, Sauer EL, Santiago O, Spencer S, Rohr JM. 2020 Divergent impacts of warming weather on wildlife disease risk across climates. Science **370**, eabb1702. (10.1126/science.abb1702) 33214248 PMC8588056

[26] Frank SA. 1996 Models of parasite virulence. Q Rev. Biol. **71**, 37-47. (10.1086/419267) 8919665

[27] Marcogliese DJ, Pietrock M. 2011 Combined effects of parasites and contaminants on animal health: parasites do matter. Trends Parasitol. **27**, 123-130. (10.1016/j.pt.2010.11.002) 21144800

[28] Hall MD, Vettiger A, Ebert D. 2013 Interactions between environmental stressors: the influence of salinity on host–parasite interactions between *Daphnia magna* and *Pasteuria ramosa*. Oecologia **171**, 789-796. (10.1007/s00442-012-2452-3) 23001624

[29] Rohr JR, Cohen JM. 2020 Understanding how temperature shifts could impact infectious disease. PLoS Biol. **18**, e3000938. (10.1371/journal.pbio.3000938) 33232316 PMC7685459

[30] Kunze C, Luijckx P, Jackson AL, Donohue I. 2022 Alternate patterns of temperature variation bring about very different disease outcomes at different mean temperatures. eLife **11**, e72861. (10.7554/eLife.72861) 35164901 PMC8846586

[31] Best A, Ashby B. 2023 How do fluctuating ecological dynamics impact the evolution of hosts and parasites? Phil. Trans. R. Soc. B **378**, 20220006. (10.1098/rstb.2022.0006) 36744565 PMC9900711

[32] Jiranek J, Miller IF, An R, Bruns E, Metcalf CJE. 2023 Mechanistic models to meet the challenge of climate change in plant–pathogen systems. Phil. Trans. R. Soc. B **378**, 20220017. (10.1098/rstb.2022.0017) 36744564 PMC9900714

[33] Graham OJ et al. 2023 Deeper habitats and cooler temperatures moderate a climate-driven seagrass disease. Phil. Trans. R. Soc. B **378**, 20220016. (10.1098/rstb.2022.0016) 36744566 PMC9900705

[34] Melero I, González R, Elena SF. 2023 Host developmental stages shape the evolution of a plant RNA virus. Phil. Trans. R. Soc. B **378**, 20220005. (10.1098/rstb.2022.0005) 36744567 PMC9979778

[35] Geffersa AG, Burdon JJ, Macfadyen S, Thrall PH, Sprague SJ, Barrett LG. 2023 The socio-economic challenges of managing pathogen evolution in agriculture. Phil. Trans. R. Soc. B **378**, 20220012. (10.1098/rstb.2022.0012) 36744561 PMC9900704

[36] Feau N, Dhillon BD, Sakalidis M, Dale AL, Søndreli KL, Goodwin SB, LeBoldus JM, Hamelin RC. 2023 Forest health in the Anthropocene: the emergence of a novel tree disease is associated with poplar cultivation. Phil. Trans. R. Soc. B **378**, 20220008. (10.1098/rstb.2022.0008) 36744569 PMC9900707

[37] Manley R et al. 2023 Conservation measures or hotspots of disease transmission? Agri-environment schemes can reduce disease prevalence in pollinator communities. Phil. Trans. R. Soc. B **378**, 20220004. (10.1098/rstb.2022.0004) 36744563 PMC9900712

[38] Manzi F, Schlösser P, Owczarz A, Wolinska J. 2023 Polystyrene nanoplastics differentially influence the outcome of infection by two microparasites of the host *Daphnia magna*. Phil. Trans. R. Soc. B **378**, 20220013. (10.1098/rstb.2022.0013) 36744559 PMC9900706

[39] Aulsebrook LC, Wong BBM, Hall MD. 2023 Can pharmaceutical pollution alter the spread of infectious disease? A case study using fluoxetine. Phil. Trans. R. Soc. B **378**, 20220010. (10.1098/rstb.2022.0010) 36744558 PMC9900710

[40] Law KL. 2017 Plastics in the marine environment. Annu. Rev. Mar. Sci. **9**, 205-229. (10.1146/annurev-marine-010816-060409) 27620829

[41] Küster A, Adler N. 2014 Pharmaceuticals in the environment: scientific evidence of risks and its regulation. Phil. Trans. R. Soc. B **369**, 20130587. (10.1098/rstb.2013.0587) 25405974 PMC4213597

[42] Fent K, Weston AA, Caminada D. 2006 Ecotoxicology of human pharmaceuticals. Aquat. Toxicol. **76**, 122-159. (10.1016/j.aquatox.2005.09.009) 16257063

[43] Richmond EK, Rosi EJ, Walters DM, Fick J, Hamilton SK, Brodin T, Sundelin A, Grace MR. 2018 A diverse suite of pharmaceuticals contaminates stream and riparian food webs. Nat. Commun. **9**, 4491. (10.1038/s41467-018-06822-w) 30401828 PMC6219508

[44] Aulsebrook LC et al. 2020 Reproduction in a polluted world: implications for wildlife. Reproduction **160**, R13-R23. (10.1530/REP-20-0154) 32442963

[45] Dobson A, Lafferty KD, Kuris AM, Hechinger RF, Jetz W. 2008 Homage to Linnaeus: how many parasites? How many hosts? Proc. Natl Acad. Sci. USA **105**, 11 482-11 489. (10.1073/pnas.0803232105) 18695218 PMC2556407

